# Predation Cues in Solitary bee Nests

**DOI:** 10.1007/s10905-017-9626-0

**Published:** 2017-06-24

**Authors:** Justyna Kierat, Michał Filipiak, Hajnalka Szentgyörgyi, Michal Woyciechowski

**Affiliations:** 1 Institute of Environmental Sciences, Jagiellonian University, Gronostajowa 7, 30-387 Kraków, Poland; 2 Department of Pomology and Apiculture, Faculty of Biotechnology and Horticulture, University of Agriculture in Kraków, Al. 29. Listopada 54, 31-425 Kraków, Poland

**Keywords:** Nesting, *Osmia bicornis*, predation, red mason bee, risk cues, solitary bees, rodents

## Abstract

Predation at the nesting site can significantly affect solitary bees’ reproductive success. We tested female red mason bees’ (*Osmia bicornis* L.) acceptance of potential nesting sites, some of which were marked with cues coming from predated conspecifics (crushed bees) or from a predator itself (rodent excreta). In our experiment, females did not avoid nests marked with either of the two predator cues. We suggest that bee females do not recognize these two cues as risky. Alternatively, costs of abandoning natal aggregation might be too high compared with any perceived predation risk of staying. Moreover, the presence of crushed bees can provide positive information about the presence of conspecifics and, possibly, information about a nesting aggregation that may be preferred by bees when choosing a nesting site.

## Introduction

Prey animals minimize the risk of predation by various types of adaptations, like morphological (e.g. shape and size changes; Tollrian 1995), physiological (e.g. toxins; Williams et al. 2003), life historical (e.g. production of diapausing forms; Ślusarczyk 2010) and behavioral ones (e.g. by diminishing activity when the probability of encountering a predator is high or by avoiding places with a high risk of being attacked; Matassa and Trussell 2011; Suselbeek et al. 2014). Information about predators can come from the predator itself (e.g. kairomones; Kobak et al. 2010) or from other prey animals. The information provided by other prey individuals can be either cues whose purpose is to warn others about the predation risk (Seyfarth et al. 1980), or cues that are only by-products of the attack, but are perceived as a risk cue, e.g. body fluids released from wounds (Czarnoleski et al. 2010). The evolution of dedicated signals that inform about risk is expected only if a signaler benefits from alerting others (directly or by helping related individuals).

Honeybee and bumblebee workers avoid flowers marked with the scent of dead individuals, and flowers on which they previously experienced unsuccessful predator attack or where another bee was temporarily captured (Abbott 2006; Dukas 2001; Llandres et al. 2013). In the latter case, the communicating substance evolved as a predator warning signal. When related females from the same colony forage in one area and encounter their kin during foraging, exchange of information between workers improves colony’s performance. Similar substances are not expected to have evolved in solitary bees, which do not establish colonies of related individuals. However, solitary bees still may react to the cues coming from other individuals killed by a predator. According to theoretical models, solitary bees are expected to accept lower risk during foraging than workers of social bees, because their death is a definite end of their reproduction, in contrast to worker bees whose fitness depends on reproduction of their kin in the colony (Clark and Dukas 1994; Rodríguez-Gironés and Bosch 2012). Thus, the response of solitary bees to predation cues in the environment should be even stronger than that of social bees. In fact, solitary bees have been shown to discover chemical and/or visual cues that signal predator presence, and to modify their behavior to reduce risk of being captured (Wcislo and Schatz 2003; Gonzálvez and Rodríguez-Gironés 2013). However, the reaction of solitary bees to predator risk cues is poorly understood, and some results are contrary to expectations, indicating that solitary bees may in fact respond to risk cues to a lesser degree than social bees (Reader et al. 2006), for example by ignoring the olfactory information about a predator (Wcislo and Schatz 2003).

The places where bees are vulnerable to predator attack are primarily foraging sites (with predators waiting for their prey on flowers, e.g. spider crabs; Ings and Chittka 2008; Reader et al. 2006) and nesting sites that can be destroyed by birds or rodents (Krunić et al. 2005). Choosing a nesting site is a particularly important decision because developing bees are confined to one place for their entire development and overwintering period (Raw 1972). Nesting aggregations of bees can persist for several seasons. It is possible that after discovering a nesting aggregation, a predator will remember the exact location and return to feed in that place again. Particularly, birds can remember locations where they recently fed and return to them (Clayton and Krebs 1994). Therefore, traces of a recent predation event can be a valuable cue for solitary bee females choosing a nesting site. Studies on bees and their invertebrate hosts focused on many aspects of their relationships, e.g. mechanisms of finding prey by parasitoids (Filella et al. 2011; Glasser and Farzan 2016) or adaptations of bees that reduce negative effect of nest parasites (Seidelmann 2006). Less is known about predation of nests by larger predators, such as birds or rodents. It is also not known whether bee females avoid nesting in places with high risk of being destroyed by such predators.

We aimed to test whether the solitary red mason bee (*Osmia bicornis* L.) avoids nesting in sites marked with scent of killed conspecifics or rodents, perceiving those sites as potentially risky and unfavorable. We also tested whether females that chose to nest in control and treated trapnests differ in size, which would indicate that female’s response to risk cue depend on her mass. Steffan-Dewenter and Schiele (2004) have shown that red mason bees are philopatric and that larger females have a higher probability of returning to their natal nest sites. Smaller females may be more likely to disperse, probably because they are outcompeted by larger females from their natal locality (Steffan-Dewenter and Schiele 2004). Antagonistic behavior, like nest usurpation, is observed between red mason bee females within an aggregation, even if the number of nesting places is not limiting. Indeed, larger females are more often successful usurpers (Kim 1997). If smaller females are normally forced to leave because of larger individuals, it may be beneficial for them to accept more risky nesting sites if there are fewer females with which they must compete. Therefore, the reaction to predator cues could be manifested by a lower proportion of returning females and/or lower mean body mass.

## Materials and Methods

Two experiments were conducted in two subsequent years. We constructed artificial trapnests to serve as nesting places for the bees. Each trapnest consisted of a PVC tube 30 cm long and 10 cm in diameter. Inside each tube were ca. 100 reed stems, open on both sides with a node in the middle. Each half of a stem was a potential nesting site for one female bee. A small PVC tube (25 cm long and 3 cm in diameter) containing red mason bee cocoons (50 male and 50 female cocoons) was placed inside the larger tube with the reed stems. The bees were sexed on the basis of morphological differences in the appearance of the head, after cutting the top of the cocoon, which is used as a standard procedure for determining sex (Seidelmann et al. 2010). Two types of trapnests were constructed: ten with predation cue (treated) and ten without it (control). In Experiment 1, twenty crushed bees were added to each trapnest in the evening before the day of placing the trapnests in the field. Ten male and ten female bees were taken out of their cocoons, frozen and crushed in a mortar with a few drops of water. The resulting macerate was smeared on the inside of the PVC pipe of trapnest before adding reed stems, and the solid parts of the crushed bees were put inside the small PVC pipe with live bee cocoons. The fresh mass of crushed bees added to treated trapnest was 1.69 ± 0.07 g (mean ± SD). In Experiment 2, the predator cue was prepared by mixing sawdust from four cages housing rodents: 3 male common voles, 3 female common voles, 2 male mice, 8 female mice. Sawdust was in the cages between 2 and 3 weeks prior to collecting and using it in Experiment 2, and at the moment of use was soaked with urine and mixed with excrements. To each of 10 treated trapnests, 0.5 l of sawdust was added, part of which was placed into the small PVC tube with the cocoons and part of which was placed into the large PVC tube with reeds. The control trapnests received the same amount of clean sawdust.

At the beginning of April, the trapnests were affixed to a tree or a bush in the meadows near the Institute of Environmental Sciences, Jagiellonian University, Kraków. In Experiment 1, the trapnests were located at least 100 m apart from each other. In Experiment 2, the paired design was applied, with one control and one treated trapnest placed less than 50 m apart, preferentially on two adjacent trees or bushes. The locations were visually assessed to be maximally similar. Pairs of trapnests were placed at least 100 m from each other. Red mason bees are gregarious and tend to return to the place from which they emerged from their cocoon to establish their own nests, referred to as philopatry (Steffan-Dewenter and Schiele 2004). This method of establishing nest aggregations was successfully applied in previous studies (Moroń et al. 2013).

The trapnests were visually monitored in the field during the day in order to make sure that the bees emerged and started their nesting activities. We checked whether the paper securing the end of the PVC tube with cocoons was torn, indicating that emerging bees had chewed their way out, and whether females were flying at the entrances of nests. The observations were done without disturbing the flying bees. A month after installation, after the controls of trapnests revealed bee nesting activity, the trapnests were collected after sunset (in Experiment 1) or shortly after dawn (Experiment 2), and each packed separately into transparent plastic bags. Female bees usually spend the nights inside their nests (Seidelmann 2006). Therefore, collecting nests after sundown or early in the morning before temperature rises, allowed us to collect all, or at least most, of the females from a nesting aggregation together with the trapnest. In Experiment 1, the trapnests were kept overnight in a climatic chamber in constant darkness at +5 °C. The next morning, they were moved to the laboratory and placed in light at room temperature. In Experiment 2, the trapnests were transferred to the laboratory immediately from the field. Bees exiting the nests were removed from the plastic bag to be sexed, counted and weighed.

The numbers of females found in the control and treated trapnests were compared with a Mann-Whitney U test in Experiment 1 and with Wilcoxon’s test in Experiment 2. The body masses of females nesting in the two types of trapnests were transformed with the natural logarithm and compared using GLM with treatment as fixed factor and trapnest nested in treatment as a random factor.

## Results

**Fig. 1:**
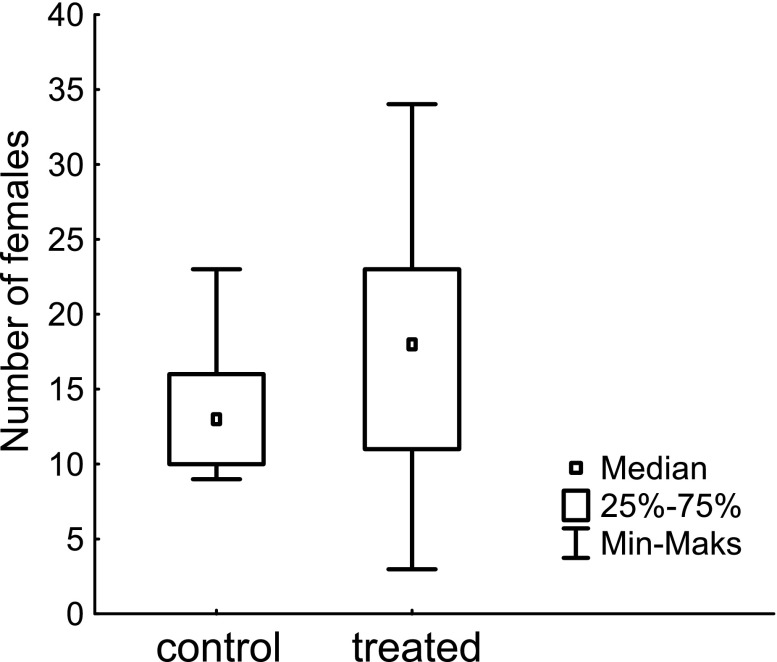
Numbers of red mason bee females nesting in control and treated with risk cue trapnests in Experiment 1. Risk cue was prepared from the crushed conspecific bees

In Experiment 1, 19 trapnests were collected from the field (one control trapnest was not found) and in total, 311 females nested in them. The numbers of females found in the control and treated trapnests did not differ significantly (U = 27.50, *p* = 0.17; Fig. 1). Although the body masses of females differed between trapnests (F_17;286_ = 1.75; *p* = 0.034), there were no differences between control and treated trapnests (F_1,286_ = 0.73, *p* = 0.40; mean ± SD: 0.099 ± 0.019 g (control), 0.101 ± 0.019 g (treatment)).

**Fig. 2:**
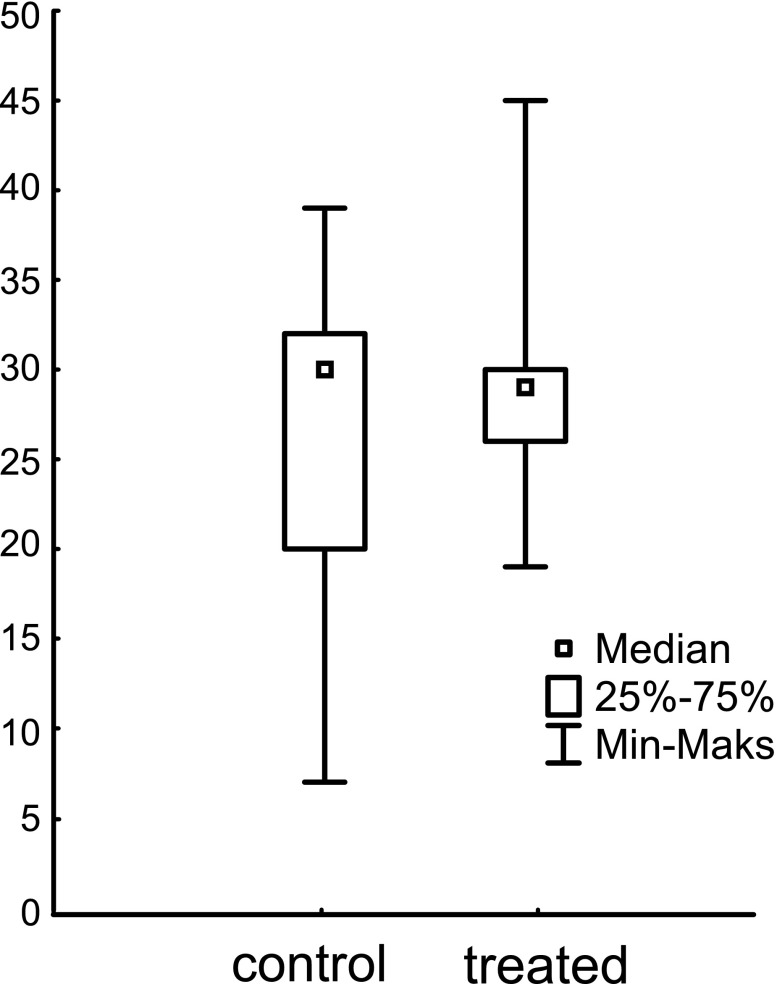
Numbers of red mason bee females nesting in control and treated with risk cue trapnests in Experiment 2. Risk cue was prepared from sawdust soaked with rodent urine

In Experiment 2, 18 trapnests (9 pairs) were analyzed (one trapnest was destroyed in the field, and the corresponding trapnest could not be used because of paired design of the experiment). In total, 484 females were collected from the trapnests. There were no significant differences in numbers of females found in control and treated trapnests (Z = 0.53, *p* = 0.59). Similarly as in Experiment 1, body masses of females differed between trapnests (F_16;456_ = 1.91; *p* = 0.017; Fig. 2), but not between treatment and control (F_1;456_ = 2.75; *p* = 0.11; mean ± SD: 0.085 ± 0.015 g (control), 0.083 ± 0.015 g (treatment)).

## Discussion

Contrary to our expectations, we did not observe preference of red mason bee females for any type of our experimental trapnests. There was also no significant difference between body masses of females nesting in treated and control trapnests. Females’ dispersal may differ according to body mass, suggesting that smaller individuals are less competitive (Steffan-Dewenter and Schiele 2004), and body size is also related to flight abilities (Seidelmann et al. 2010). That might result in small females being more likely to stay in a suboptimal endangered nesting site. However, this was not the case in our experiment.

There are several possible explanations for our results. First of all, the females may have simply not been able to detect the signals indicating predation event. We marked the trapnests with cues immediately before installing them in the field, and up to the emergence of the females, the cues could have lost relevant olfactory properties. The signal could also have been difficult to detect if it was too weak. However, as each trapnest in Experiment 1 was marked with 20 bees, and their tissues were smeared at the entrances of trapnests, it was probably sufficient for the females to detect. The remnants of crushed bees were also added to the tubes with cocoons, and emerging individuals were exposed to them. Likewise, in Experiment 2, the sawdust with rodents’ smell was added to the tubes with cocoons and mixed with reed straws inside the trapnest. Bee females nesting in pre-existing cavities visit and examine several nests before choosing one (Levin 1966; Ramos et al. 2010; Raw 1972), and it was unlikely that females examining reed stems in our treated trapnests did not discover the cue. Presence of dead bees in Experiment 1 gave additional possibility to visually detect the risk cue. However, although bees can well recognize objects on the basis of colour and/or shape (Ings and Chittka 2008), darkness inside the nest traps made olfactory cues more probable for bees to use.

Red mason bee females as well as many other solitary bees are gregarious (Michener 2007). Several benefits from gregarious nesting have been suggested, e.g. more effective protection against nest parasites, decreased cost of searching for suitable nesting sites, or reuse of old nests (Rosenheim 1990). Philopatry causes young females return to their natal nesting place (Steffan-Dewenter and Schiele 2004), and may assure the perpetuation of an aggregation that can grow with each season. However, females are also attracted to odors that signal the presence of an existing aggregation, such as odor of empty cocoons (Pitts-Singer 2007; Artz et al. 2013). That preference is quite strong: nests treated with ethanol extract of empty cocoons attracted substantially more nesting *O. lignaria* females, compared with untreated nests in the same row of trees in an almond orchard (M. Allan, pers. comm.). If the presence of other bees is more important than the risk of future predation, then the crushed bees placed in trapnests could have been not only a risk cue for bees, but also a conspecific recognition cue. In that case, the two different meanings of that signal could have cancelled each other or been weighed against other factors such as nest availability. Furthermore, the presence of bee body parts in the pipe with the cocoons may have been similar to body parts that remain when bees chew through dead or slow siblings upon emergence from natal nests, which contain a linear series of cells. As such, the odor of body parts may indicate that successful nesting occurred in those nesting sites rather than signaling the loss of bees to predators. Any attractiveness of rodent scent is more difficult to explain. Using empty rodent burrows as nesting sites is a frequent case in bumblebees (Michener 2007), but there are no data in the literature about their use by *Osmia* bees. It is possible that bees ignored olfactory cues in the absence of any corresponding visual cues (Wcislo and Schatz 2003).

In solitary bees that nest in pre-existing cavities, the availability of nesting sites can be a limiting factor. A female that already found a suitable nest cavity may decide to stay in it, even though it is not optimal, e.g. if it is associated with a predation risk. Most of trapnests in Experiment 1 were placed no more than 300 m from the nearest control trapnest. It is a distance possible to traverse by red mason bee females. They have been reported to return to their nests even from a distance of 500 m (Gathmann and Tscharntke 2002). Our experimental bees could have failed to find other trapnests and return to their natal site, however, in that case at least some of the dispersing females would probably establish nests in natural cavities or die in the meantime. It is more likely that females decided to nest at their site of emergence because the cost of abandoning it and searching for another one was too high. This explanation for lack of preference does not held for Experiment 2, where control and treated trapnest were placed close to each other. The cost of switching between the two trapnests would be very small and if female bees did not do it, the treated trapnests were probably no less attractive than control ones.

Females of solitary bees use a number of environmental cues to select the most suitable nesting site (Budriene et al. 2004; Potts and Willmer 1997). However, our study indicates that cues from dead conspecifics and rodents are not important predator cues for the red mason bees. We suggest that females did not respond to predation cue or at least not to the ones chosen for this study, because it lacked meaning or they are so conservative in their initial choice of nesting site that they do not abandon their natal place even in the presence of risk cues.
